# Television, physical activity, diet, and body weight status: the ARIC cohort

**DOI:** 10.1186/1479-5868-5-68

**Published:** 2008-12-17

**Authors:** Anne-Marie Meyer, Kelly R Evenson, David J Couper, June Stevens, Mark A Pereria, Gerardo Heiss

**Affiliations:** 1 Department of Epidemiology, University of North Carolina at Chapel Hill, Chapel Hill, NC, USA; 2 Department of Nutrition, University of North Carolina at Chapel Hill, Chapel Hill, NC, USA; 3 Department of Biostatistics, University of North Carolina at Chapel Hill, Chapel Hill, NC, USA; 4 Department of Epidemiology, University of Minnesota, Minneapolis, MN, USA

## Abstract

**Background:**

Television (TV) watching is the most common leisure activity in the United States. Few studies of adults have described the relationship between TV and health behaviors such as physical activity, diet, and body weight status.

**Methods:**

Extant data from the Atherosclerosis Risk in Communities (ARIC) Study were analyzed to assess the association of TV with physical activity, diet, and body mass index (BMI) among 15,574 adults at baseline (1986–89) and 12,678 adults six years later. Television, physical activity, and diet were collected with questionnaires and BMI was measured at both time points. Based on baseline TV exposure, adults were categorized into high, medium, and low TV exposure. Linear and logistic regression models were adjusted for gender, age, race-center, smoking, education, and general health.

**Results:**

Relative to participants who had low TV exposure, those with high TV exposure were more likely to be less physically active and have a poorer dietary profile at baseline and six-years later. Participants with high TV exposure at baseline had a 40% and 31% greater odds of being considered insufficiently active at baseline (1.40, 95% CI 1.26, 1.55), and six years later (1.31, 95% CI 1.18, 1.46). At baseline, high TV exposure was also associated with a 20% to 30% greater odds of being above the median for servings of salty snacks (1.37, 95% CI 1.24, 1.51), sweets (1.26, 95% CI 1.15, 1.38), and sweetened drinks (1.29, 95% CI 1.17, 1.42), and below the median for fruit and vegetable servings (1.36, 95% CI 1.24, 1.50). Higher TV exposure was also cross-sectionally associated with a greater odds for being overweight or obese (1.43, 95% CI 1.29, 1.58). Similar associations were observed between baseline TV exposure and six-year physical activity and diet, but were not observed with BMI after six years follow-up.

**Conclusion:**

These results support the hypothesis that time spent watching TV is associated with deleterious effects on physical activity, diet, and BMI.

## Background

Over the last half century, television (TV) has become the most popular leisure activity in the United States [1,2]. American adults watch an average of four to five hours of television each day [3]. The increase in the number of televisions per person and hours of television watching in the United States has paralleled the increase in obesity during the last fifty years [4,5]. Despite the prevalence of this sedentary behavior, there has been little research in adults that describes the relationships between television watching and physical activity, diet, and obesity.

The sparse research on television with physical activity or diet has produced mixed results. The studies on television and physical activity have primarily been cross-sectional and the reported associations have been low (unadjusted correlations, -0.03 to -0.11) [4,6-10]. The studies on diet and television have also primarily been cross-sectional in nature, but have shown a consistent relationship between increasing television watching and poor dietary choices. In both the Nurses' Health Study and Health Professionals Follow-up Study, higher television exposure was cross-sectionally associated with diets higher in saturated fats, greater servings of red and processed meat, french fries, refined grain, snacks, sweets or desserts, and fewer servings of fish, fruit, vegetables, and whole grains [4,7]. Neither of these cohort studies examined the relationships between television, physical activity, or diet prospectively.

Increased risk for overweight or obesity associated with television watching has been examined in several cohort and cross-sectional studies [4,6,11-21]. Higher television watching was associated with higher body weight status or skinfolds in several studies including the United States, European, Australian and American Indian populations [6,15,16,19-23]. In the Nurses' Health Study and Health Professionals Follow-up Study, prospective analyses found an increased risk of obesity associated with higher television watching [4,12-14]. This increased risk was independent of physical activity. However, none of the previous studies examined body weight status while considering all three behavioral risk factors, TV, physical activity, and diet. Since no studies have been published on the relationships between television, physical activity, diet, and body weight status in the same cohort, either cross-sectionally or prospectively, this study sought to explore the association of television watching with each risk factor using a cohort design.

## Methods

### Study population

The Atherosclerosis Risk in Communities (ARIC) Study was designed to examine risk factors for cardiovascular disease and its related morbidity and mortality. Participants were selected from four US communities: Washington County, Maryland; northwest suburbs of Minneapolis, Minnesota; Jackson, Mississippi; and Forsyth County, North Carolina. A probability sample of men and women ages 45 to 64 years was recruited from each community, from which 15,792 adults completed the first clinic visit. The first clinic visit occurred between 1987 and 1989 and included a physical examination and an interview by trained personnel regarding medical history, health status, diet, and physical activity. Clinic visits were repeated at approximately three-year intervals. Further details on the ARIC cohort are available elsewhere [24,25].

### Television and physical activity measurement

The Baecke physical activity questionnaire was designed to study habitual physical activity and distinguish between different dimensions of physical activity using semi-continuous indices of sport, leisure, and work activity [26]. The questionnaire was interviewer administered at the baseline (1986–1989) and third clinic visit (1993–1995) of the ARIC study. The questionnaire included an item on television watching as part of the leisure activity index. This subjective question of television exposure asked: "During your leisure time do you watch television?" and allowed five responses: never, seldom, sometimes, often, and very often. The answers were ranked on an ordinal scale from 1 (low) to 5 (high). From this ordinal scale, television watching was collapsed into three exposure levels: low (never/seldom, n = 2878), medium (sometimes, n = 7293), and high (often/very often, n = 5403). Because television watching is a component question of the leisure index, the remaining three questions on leisure activity were analyzed separately. These items included information on walking and biking and were ordinally scaled from five responses (never, seldom, sometimes, often, and very often). The third question asked about minutes spent walking or biking to and from work or shopping and respondents could answer < 5 minutes/week, 5–<15 minutes/week, 15–<30 minutes/week, 30–<45 minutes/week, or ≥ 45 minutes/week.

Sport and work physical activity were assessed with semi-continuous indices created from individual component questions and have been described in detail elsewhere [26-28]. Using the reported activities from the sport index, participants were also dichotomised as sufficiently or insufficiently active. Sufficient activity was defined as regular physical activity, or participation in at least one hour per week of activity for 10 or more months of the year [27,28]. Insufficient activity was identified by less than one hour per week and/or less than 10 months per year.

The reliability and validity of the Baecke questionnaire have been examined in both European and American populations. Test-retest reliability correlations of total physical activity from the three indices range from 0.65 to 0.93 [26,29-31]. Validation of the Baecke has been conducted using physical activity diaries, maximum oxygen consumption, accelerometers, and doubly-labeled water [29,30,32-39]. In a doubly-labeled water study of three physical activity questionnaires, the Baecke had the highest agreement for total physical activity (Pearson's r = 0.69) [40].

### Diet measurement

Usual dietary intake was collected at baseline and at the third clinic visit using a semi-quantitative, interviewer-administered food frequency questionnaire (FFQ). The ARIC FFQ contains 66-items and was based on the original Willett 61-item FFQ [41]. The ARIC FFQ is organized into seven sections; 1) dairy, 2) fruit, 3) vegetables, 4) meats and fish, 5) sweets and baked goods, 6) miscellaneous, carbohydrates, fried foods, and 7) beverages. Interviewers asked participants; "In the last year how often, on average, did you consume ______?" Nine responses were available for each food item ranging from "almost never" to "more than 6 times per day". Each response was assigned a weight to estimate servings per day and daily intake of nutrients [42].

Using the standard serving sizes from the FFQ, daily servings of each food item were calculated and summed to create food groups. Dietary outcomes were categorized into the following food groups: fruit, vegetables, fruit and vegetables combined, salty snacks, sweets, sweetened drinks [43]. Total caloric intake, total fat and percent saturated fat, and estimated nutrient values were calculated at the Channing Laboratory, Harvard Medical School.

The original Willett FFQ has been validated in a number of populations and is a well-recognized dietary tool with validity correlations between 0.35 and 0. 74 [41,44,45]. The reliability of the ARIC FFQ has been estimated among both white and African Americans participants in the ARIC study [46]. Median reliability coefficients were 0.48 and 0.63 for White women and men, respectively, and 0.45 and 0.50 in African American women and men, respectively.

### Measurement of body weight status

Anthropometric measurements of weight and height were obtained during both clinic visits and were used to calculate body mass index (BMI) (weight in kilograms divided by height in meters squared). Four weight status groups were created using BMI: underweight (<18.5 kg/m^2^), normal weight (≥ 18.5–<25 kg/m^2^), overweight (≥ 25–<30 kg/m^2^), and obese (≥ 30 kg/m^2^) [47].

### Measurement of other study variables

At baseline, participants reported cigarette smoking (current, former, never) and years of education (<high school, high school, some college, or higher). Also at baseline, participants were asked to rank their general health as excellent, good, fair, or poor. Because one of the study centers was entirely African American (Jackson, MS) and others predominately white, a race-center variable to control for effects of race and center was created by combining each participant's race with their respective study center (e.g., Forsyth County African American; Forsyth County white).

### Statistical analysis

In order to examine the associations between television, physical activity, diet, and body weight status participants were excluded who did not answer the television question at baseline (n = 32) or were missing information on age, race, gender, body mass index, general health status, or smoking (n = 83). Additionally, in order to control for the effect of race and center, individuals other than white or African American and all non-white participants from Minneapolis and Washington were excluded (n = 103). Excluded from the prospective analysis were those individuals who died (n = 722) or did not return to the third clinic visit (n = 2,125), as well as those missing information on both physical activity and diet outcomes at the third clinic visit (n = 49). Final sample sizes were 15,574 for the cross-sectional analysis and 12,678 for the prospective analysis, the latter referred to as "the cohort".

The associations of baseline television watching with baseline and six-year physical activity, diet, and body weight status were estimated from multivariable linear and logistic models. Analysis of variance (ANOVA) was used to estimate the mean of each outcome variable at each of the three levels of television exposure (low, medium, high). For the linear models, analyses were also performed using log transformations of all non-normally distributed outcomes. The results were unchanged using log-transformed variables and therefore, for ease of interpretation, they were left in their original state. For logistic models, outcomes were dichotomized as: having a BMI >= 25, being below the median for positive outcomes (e.g., physical activity), or above the median for negative outcomes (e.g., total energy intake). Low television exposure was defined as the referent and the odds of each outcome were estimated for medium and high television exposure.

To assess the temporal association of baseline television watching with physical activity and diet over the six years of follow-up, the baseline value of each outcome was included in all prospective models for a further "baseline-adjusted" model. In a longitudinal analysis, adding the baseline outcome variable to the model will adjust for the cross-sectional associations at baseline between television and each outcome of interest.

All statistical models included the following covariates measured at baseline: age, race-center, gender, BMI, education, smoking, and general health, with categorization indicated in Table 1. An *a priori *conceptual model based on a literature review and previous ARIC analyses helped direct model building with regard to confounder/covariate cut-points, television categories, and outcome categorizations. Post-hoc analyses were performed to assess if different outcome cut-points (median, vs. mean dichotomization; tertile vs. quartile categorization) resulted in different associations or conclusions. Analyses were also performed to assess any effect of outliers (defined as ≥ 3 standard deviations from the mean) and missing data. The statistical models, measures of effect, and 95% confidence intervals were computed using SAS V9.1 (Cary, NC; 2002).

**Table 1:** Selected baseline covariates* by television exposure from the analysis sample, n = 15,574.

	**Low TV exposure**		**Medium TV exposure**		**High TV exposure**	
	
	**N**	**%**	**N**	**%**	**N**	**%**
	
**Age (years)**						
45–49	886	30.8	1938	26.6	1341	24.8
50–59	1415	49.2	3770	51.7	2666	49.3
>= 60	577	20.1	1585	21.7	1396	25.8
**BMI**						
Under weight	26	0.9	54	0.7	62	1.2
Normal weight	1131	39.3	2408	33.0	1475	27.3
Overweight	1087	37.8	2853	39.1	2182	40.4
Obese	634	22.0	1978	27.1	1684	31.2
**Race**						
White	2415	83.9	5439	57.6	3569	66.1
African American	463	16.1	1854	42.4	1834	33.9
Gender						
Male	1167	59.4	3090	42.4	2720	50.3
Female	1711	40.6	4203	57.6	2683	49.7
**Center**						
Jackson, MS	415	14.4	1683	23.1	1573	29.1
Washington County, MD	765	26.6	1903	26.1	1286	23.8
NW suburbs of Minneapolis, MN	931	32.4	1792	24.6	1236	22.9
Forsyth County, NC	767	26.6	1915	26.3	1308	24.2
**General Health Status**						
Excellent	1240	43.1	2466	33.8	1464	27.1
Good	1269	44.1	3484	47.8	2532	46.9
Fair	322	11.2	1139	15.6	1127	20.9
Poor	47	1.6	204	2.8	280	5.2
**Education**						
Less than high school	497	17.3	1646	22.6	1550	28.7
At least high school education, but	1131	39.3	2987	40.9	2236	41.4
less than college						
College education or higher	1250	43.3	2660	36.5	1617	29.9
**Smoking Status**						
Current	588	20.4	1792	24.6	1392	31.3
Former	941	32.7	2325	31.9	1757	32.5
Never	1349	46.9	3176	43.6	1954	36.2

The analysis for this study was approved by the Public Health-Nursing IRB at the University of North Carolina at Chapel Hill, and participants provided written, informed consent at each ARIC center.

## Results

At baseline, participants were predominately white (73%) and most (76%) reported completing at least a high school education (Table 1). While the majority of the sample (67%) was overweight or obese, approximately 80% reported "good" or "excellent" general health. Fewer than one-third of the participants were current smokers. More than one-third of the sample reported watching television "often" or "very often" (Table 2). The majority of the study population (>60%) was insufficiently active at both time points. In the prospective cohort (n = 12,678), the median number of servings of fruits and vegetables over the six years increased from 3.7 to 4.1 servings per day, while the servings of salty snacks, sweets and sweetened beverages remained relatively constant. Total energy intake and total fat both declined for the cohort, while sport activity increased slightly.

**Table 2:** Description of television watching, physical activity, and dietary outcomes at baseline and six years later.

	**Baseline n = 15,574**		**Follow-up n = 12,678**	
	
	**Median**	**Range**	**Median**	**Range**
	
Sport index	2.3	1.0 – 5.0	2.5	1.0 – 5.0
Work index among workers*	2.6	1.0 – 4.9	2.6	1.0 – 5.0
Total caloric intake (kcal)	1530	500 – 4192	1494	504 – 4181
Total fat (grams)	55.4	5.4 – 235.1	50.9	6.7 – 228.0
Percent kcal from total fat (%)	33.1	5.9 – 62.6	31.3	6.4 – 61.8
Percent kcal from saturated fat (%)	12.0	1.3 – 29.0	11.3	1.6 – 27.7
Fruit & vegetables combined (servings per day)	3.7	0 – 53.0	4.1	0 – 69.0
Salty snacks (servings per day)	0.21	0 – 6.5	0.21	0 – 8.5
Sweets (servings per day)	1.0	0 – 27.4	1.0	0 – 37.0
Sweetened drinks (servings per day)	0.1	0 – 11.0	0.1	0 – 12.0
Body mass index	26.9	14.2 – 65.9	27.5	13.2 – 60.2
	
	**n^§^**	**%**	**n^§^**	**%**
	
**Television**				
Never	296	1.9	255	2.0
Seldom	2582	16.6	1825	14.4
Sometimes	7293	46.8	5912	46.6
Often	4133	26.5	3735	29.5
Very often	1270	8.2	951	7.5
**Sufficient physical activity**				
Insufficient	10681	68.7	8019	63.5
Sufficient	4856	31.3	4608	36.5
**Leisure walking**				
Never or seldom	4591	29.5	3032	23.9
Sometimes	7539	48.4	6221	49.0
Often or very often	3444	22.1	3424	27.0
**Obesity**				
Underweight	142	0.9	115	0.9
Normal	5014	32.2	3510	27.7
Overweight	6122	39.3	4981	39.3
Obese	4296	27.6	4058	32.0

* Among those who worked outside of the home (full or part-time n = 7912)

^§ ^Due to missing data, N does not always add up to total sample size

Participants who did not return for the third clinic visit (n = 2,125) or died (n = 722) during follow-up were more likely to watch television "often" or "very often" at baseline than individuals who remained in the study (42% vs. 33% respectively). They were also significantly (p < 0.001) more likely to have a lower sport index (2.31 vs. 2.46), work index (2.10 vs. 2.20), and consume fewer fruits (1.90 vs. 2.03 servings), salty snacks (0.35 vs. 0.41 servings), and more sweetened drinks (0.65 vs. 0.54 servings), at baseline than those who remained in the analysis. They were more likely to smoke (40% vs. 23%), report poor health status (8% vs. 2%), and have less than a high school education (39% vs. 20%).

### Cross-sectional association with television watching

At baseline, watching television "often" or "very often" (high exposure) and "sometimes" (medium exposure) was associated with a 40% and 20% greater odds of being insufficiently active, respectively, compared to watching television "never" or "seldom" (low exposure) (Table 3). High television exposure was also associated with a 50% greater odds of being below the median of the sport index, but it was not associated with the work index. Participants with low television exposure had almost two-fold greater odds (1.96, 95% CI 1.78, 2.17) of walking during leisure time compared to those with medium television exposure. High television exposure was also associated with less walking and biking during leisure and for transportation. Increasing television exposure exhibited a graded relationship with unhealthy dietary choices (Table 3). High television exposure was associated with an approximate 20 to 30% greater odds of being above the median for servings of salty snacks, sweets, and sweetened drinks, total fat, and percent of calories from fat and saturated fat, and below the median for fruit and vegetable servings.

**Table 3:** Odds ratios (OR) and 95% confidence intervals (CI) for the association of television exposure with physical activity and diet at baseline, n = 15,574.

	**Medium TV exposure**		**High TV exposure**	
	
	OR*	**95% CI**	OR*	**95% CI**
	
Sport index^†^	1.15	1.05, 1.27	1.50	1.36, 1.66
Work index^†^	1.03	0.93, 1.15	1.05	0.94, 1.18
Leisure walking^§^	1.96	1.78, 2.17	1.60	1.44, 1.78
Leisure biking^§^	1.38	1.12, 1.71	1.29	1.02, 1.62
Minutes of walking and biking for transportation^§^	1.04	0.93, 1.17	1.37	1.21, 1.56
Leisure sweating	1.43	1.27, 1.61	1.22	1.08, 1.38
Insufficient activity	1.2	1.09, 1.31	1.40	1.26, 1.55
Fruit & vegetable servings^†^	1.18	1.08, 1.29	1.36	1.24, 1.50
Salty snack servings^‡^	1.19	1.09, 1.30	1.37	1.24, 1.51
Sweet servings^‡^	1.12	1.03, 1.22	1.26	1.15, 1.38
Sweetened drink servings^‡^	1.17	1.07, 1.28	1.29	1.17, 1.42
Total calories^‡^	0.93	0.85, 1.01	1.05	0.95, 1.15
Total fat^‡^	1.02	0.93, 1.11	1.16	1.05, 1.27
Percent kcal from fat^‡^	1.14	1.04, 1.25	1.22	1.11, 1.34
Percent kcal from saturated fat^‡^	1.10	1.01, 1.20	1.17	1.06, 1.28

* All models adjusted for baseline values of age, BMI, gender, education, general health, smoking, and race-center using indicator variables with categories

^† ^Below median

^‡ ^Above median

^§ ^Often, very often (high) vs. never, seldom, sometimes (low)

Referent: low television exposure

**Figure 1:**
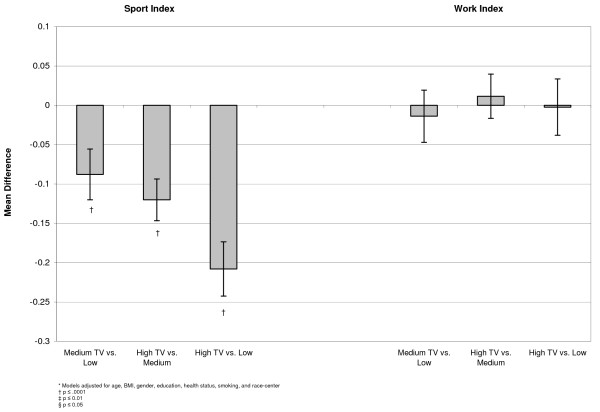
Adjusted* mean differences in physical activity by TV exposure, at baseline, n = (15,574).

**Figure 2:**
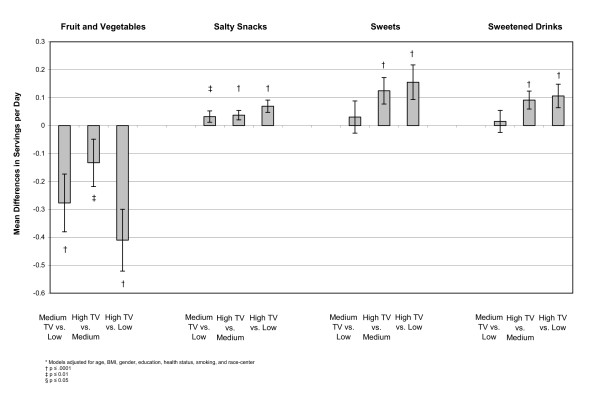
Adjusted* mean differences in fruit and vegetable, salty snack, sweets, and sweetened drink servings per day by TV exposure, at baseline, (n = 15,574).

**Figure 3:**
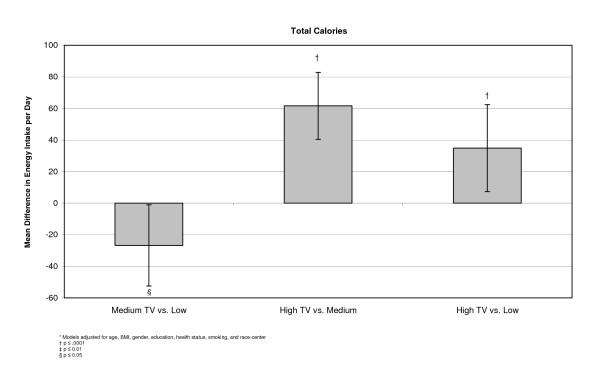
Adjusted* mean differences in total calories (kcal) by TV exposure, at baseline, (n = 15,574).

**Figure 4:**
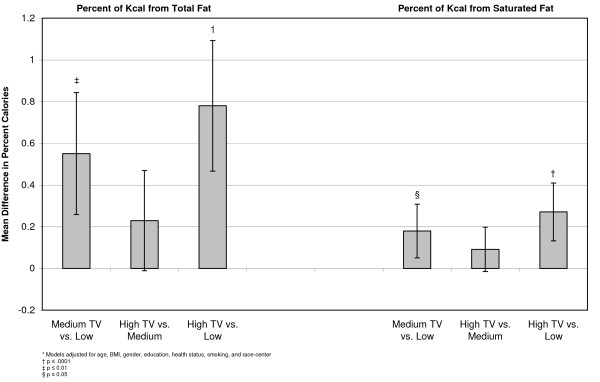
Adjusted* mean differences in percent of calories from fat and saturated fat by TV exposure, at baseline, (n = 15,574).

In a linear model, differences were examined between the adjusted means of each outcome at the three levels of exposure. These models indicated that individuals with higher television exposure participated in less sport physical activity than people who were exposed to medium or low amounts of television. There was no apparent relationship between television exposure and physical activity from the work index (Figure 1). Examining diet, we found that participants who reported high television exposure ate more servings per day of salty snacks (0.07, 95% CI 0.04, 0.09), sweets (0.16, 95% CI 0.10, 0.22), sweetened drinks (0.11, 95% CI 0.07, 0.15), percent of calories from fat (0.78, 95% CI 1.09, 0.47), and percent of calories from saturated fat (0.27, 95% CI 0.47, 0.13) than people who reported low television exposure (Figures 2, 3, 4). Those with high exposure also consumed almost one-half serving fewer fruits and vegetables per day (-0.41, 95% CI -0.52, -0.30) (Figure 2).

**Figure 5:**
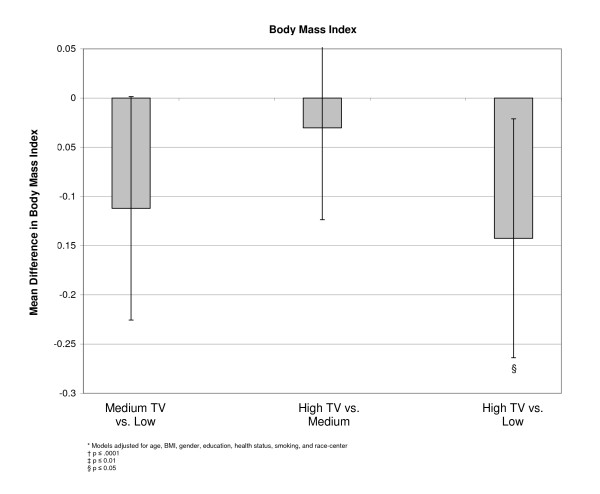
Adjusted* mean differences in body mass index by TV exposure, at baseline, (n = 15,574).

Similar relationships were observed between increasing television watching and body weight status. At baseline, participants with medium and high television exposure had a 22 and 43 percent greater odds of being overweight or obese than individuals with low exposure (Table 4). However the absolute magnitude of effect was less dramatic when comparing adjusted means in a linear model, and the only significant comparison was between low and high television exposure (Figure 5). Further adjustment for sport physical activity and total kilocalories had no appreciable affect on these results.

**Table 4:** Adjusted odds ratios (OR) and 95% confidence intervals (CI) for association of body weight status with television exposure, at baseline and at follow-up six-years later.

	**Medium TV exposure**		**High TV exposure**	
	
	**OR**	**95% CI**	**OR**	**95% CI**
	
Adjusted for covariates*				
Overweight or obese at baseline	1.22	1.12, 1.34	1.43	1.29, 1.58
Overweight or obese at follow-up	1.14	1.04, 1.25	1.16	1.05, 1.27
Adjusted for sport index^§^, total kcals^§^, and covariates*				
Overweight or obese at baseline	1.21	1.10, 1.33	1.40	1.27, 1.56
Overweight or obese at follow-up	1.13	1.04, 1.24	1.13	1.03, 1.25
Adjusted for baseline sport index^§^, total kcals^§^, BMI, and covariates*				
Overweight or obese at follow-up	1.03	0.92, 1.15	0.93	0.83, 1.04

* Covariates included baseline age, gender, education, health status, smoking, and race-center coded as indicator variables

^§ ^Baseline value of variable

Referent: low television exposure

### Prospective association with television watching

Compared with low exposure, high and medium exposure to television remained a predictor of physical activity, diet, and body weight status over the six years of follow-up (Table 4, 5). Although the six-year associations were attenuated compared to the baseline associations, a consistent relationship was still observed between higher television exposure and more unhealthy physical activity and dietary outcomes. Adjusting for the baseline association of exposure and outcome in the models resulted in further attenuation of these associations, although a significant effect was observed for most physical activity and dietary outcomes.

**Table 5:** Adjusted* odds ratios (OR) and 95% confidence intervals (CI) for association of baseline television exposure with physical activity and diet six years later, n = 12,678.

	**Medium TV exposure**		**High TV exposure**	
	
	OR*	**95% CI**	OR*	**95% CI**
	
Sport index^‡^	1.16	1.05, 1.28	1.50	1.34, 1.66
Work index^‡^	1.21	1.07, 1.37	1.20	1.05, 1.38
Leisure walking^#^	1.38	1.24, 1.54	1.44	1.28, 1.62
Leisure biking^#^	1.25	0.99, 1.57	1.47	1.12, 1.92
Minutes of walking and biking for transportation^#^	1.13	1.00, 1.28	1.28	1.11, 1.46
Leisure sweating^#^	1.14	1.01, 1.29	1.24	1.09, 1.42
Insufficient activity	1.10	1.00, 1.22	1.31	1.18, 1.46
Fruit and vegetable servings^‡^	1.21	1.10, 1.33	1.34	1.21, 1.49
Salty snack servings^§^	1.11	1.00, 1.22	1.24	1.11, 1.38
Sweet servings §	1.06	0.96, 1.17	1.23	1.11, 1.36
Sweetened drink servings §	1.18	1.07, 1.30	1.31	1.18, 1.46
Total calories §	0.96	0.87, 1.05	1.08	0.97, 1.20
Total fat §	1.06	0.96, 1.17	1.20	1.08, 1.33
Percent kcal from fat §	1.08	0.98, 1.20	1.20	1.08, 1.34
Percent kcal from saturated fat §	1.16	1.05, 1.28	1.26	1.14, 1.40
**Baseline-adjusted^†^**				
Sport index^‡^	1.13	1.02, 1.25	1.35	1.21, 1.51
Work index^‡^	1.27	1.10, 1.46	1.23	1.05, 1.43
Leisure walking^#^	1.19	1.06, 1.33	1.30	1.15, 1.47
Leisure biking^#^	1.17	0.92, 1.49	1.43	1.09, 1.88
Minutes of walking and biking for transportation^#^	1.13	1.00, 1.28	1.23	1.08, 1.41
Leisure sweating^#^	1.04	0.91, 1.18	1.18	1.02, 1.36
Insufficient activity	1.06	0.95, 1.17	1.22	1.08, 1.36
Fruit and vegetable servings^‡^	1.15	1.04, 1.28	1.23	1.09, 1.37
Salty snack servings^§^	1.06	0.95, 1.17	1.13	1.01, 1.27
Sweet servings^§^	1.02	0.92, 1.13	1.12	1.00, 1.25
Sweetened drink servings^§^	1.12	1.01, 1.25	1.21	1.08, 1.36
Total calories^§^	0.99	0.89, 1.10	1.06	0.94, 1.19
Total fat^§^	1.08	0.97, 1.21	1.15	1.02, 1.29
Percent kcal from fat^§^	1.08	0.96, 1.21	1.15	1.02, 1.29
Percent kcal from saturated fat §	1.07	0.96, 1.19	1.17	1.04, 1.31

* All models adjusted for baseline values of age, BMI, gender, education, health status, smoking, and race-center coded as indicator variables.

^† ^Baseline-adjusted models include the baseline value of the outcome variable.

^‡ ^Below median.

^§ ^Above median.

^# ^Often, very often (high) vs. never, seldom, sometimes (low).

Referent: low television exposure

The associations between television watching and body weight status were almost the same for both medium and high television exposures in the prospective model (Table 4). However, both exposure levels were still associated with greater odds of being overweight or obese at six-years follow-up relative to low exposure. Additionally, when the baseline term for body weight status was added, the prospective association attenuated to the null.

Post-hoc exploratory analyses were conducted to examine the effect of different cut-points and model choices. Regardless of the model choice (linear versus logistic model), consistent associations were observed between television and each outcome using different exposure and outcome categorizations (continuous, tertile, quartile, different dichotomization). Even when outliers were removed, the conclusions were the same (data not shown). Lastly, we also restricted the sample to only participants with complete data however the conclusions were again the same (data not shown).

## Discussion

At baseline, graded relationships were observed between television exposure, level of physical activity, diet, and body weight status. Similar relationships for physical activity and diet were still apparent in prospective analyses, although attenuated after controlling for the baseline association between television and each outcome. A different pattern was observed with television watching and body weight status, where including baseline outcome in the model eliminated any prospective effect. Adjusting for baseline in the prospective models helps us examine the temporal sequence between exposure and outcome over the six years. These adjusted models suggest that television is perhaps associated with negative behavioral choices over time.

These results also provide some evidence in support of the 'displacement hypothesis', which holds that sedentary activities, such as television watching, are substituted for more active pursuits. Because many adults have only a few hours daily for discretionary activities [1,2,48], watching television during free time may displace exercise or physically active leisure pursuits. An inverse association was observed between television exposure and each type of physical activity (i.e., sport, work, leisure walking, or leisure biking). If high exposure to television encourages people to expend less energy in other aspects of their daily lives, then the chances of these individuals meeting the recommended guidelines for physical activity are reduced.

Although the magnitude of the observed associations between television and diet appear small, the impact on the population could be significant. Results from the cross-sectional multivariable linear models indicated that adults with high television exposure consumed approximately one-half serving of fruits and vegetables less per day than those with low exposure and had higher energy intake and fat intake (data not shown). If these differences occur daily, with no additional dietary changes, they would project to a yearly burden of thousands of additional calories and hundreds of grams of fat.

Previous literature has shown that television may impact risk of chronic disease, independent of physical activity [4,7,13]. The Health Professionals Follow-up and Nurses' Health studies have identified significant associations between television watching and biomarkers for cardiovascular disease, such as low density lipoprotein, high density lipoprotein, leptin, as well as higher risk of becoming overweight and developing type 2 diabetes [4,12-14]. Television watching in this study was also significantly association with overweight and obesity. Similar to previous studies, this association appeared to be independent of physical activity and diet. The results from this study also suggest that television watching may influence other chronic disease risk factors such as diet and physical activity. It has become increasingly apparent that sedentary behaviors (such as television watching) may play an important role in physiology, health outcomes, and even gene expression [4,12,13,49-51].

### Limitations

This study is the first large cohort analysis to examine the associations of reported television exposure with physical activity, diet, and body weight status. However, it is important to recognize that the question measuring television exposure was subjective and semi-quantitative, and could result in misclassification. Additionally, although the Baecke questionnaire has shown validity and reliability in other populations [29,39,40] the television question used in this analysis has not been validated as a single item, to our knowledge. Although better measurement tools have been developed and great strides have been made in physical activity epidemiology, [52] no tool has been designed to adequately measure sedentary behaviors [53]. The television question in the ARIC study is also not an absolute measure of time, and is a subjective measure of exposure. To minimize this limitation, ancillary data was obtained (M. Hulens, personal communication, 2004 [54]) which compared the Baecke television question with a second, concurrent report of the continuous number of hours of television exposure in a Belgian population. Agreement between continuous hours of television and the single item Baecke was acceptable (chi square 92.3, p < 0.0001).

Food frequency questionnaires like the ARIC-FFQ collect information on a limited number of food items and do not assess total energy intake; therefore, these dietary outcomes contain measurement error. Yet until better methods are developed, FFQs are a practical method for gathering information on dietary behaviors in large cohort studies [44]. Although validation of the ARIC FFQ is not available, the Willet FFQ, on which the ARIC questionnaire is based, has been validated and used in other well-recognized cohort studies [41,55,56]. Reliability of this tool has been shown to be between 0.45 – 0.63 across visits (3 years) in this study population [46]. Future studies could consider exploring measures of diet quality, in addition to the components that we assessed.

Another limitation of this study is the loss of participants between visits. These individuals (n = 2,847) were less healthy, reported higher television exposure, and had more negative diet and physical activity patterns than those who remained in the study. Because these individuals fall into both the high exposure and more unhealthy outcome categories at baseline, it is unlikely that the results would be different had these participants remained in the study.

This study is observational and relies on recall of both exposure and outcomes. Therefore, in a study of this nature it is impossible to untangle any effect of previous television, activity, and dietary behaviors from our observations. Additionally, baseline data collection began in the mid-eighties and societal television, physical activity, and diet behaviors will not be the same as present day. It is important to recognize that the generalizability of this study to current sedentary behaviors and other populations is limited.

Additionally, physical activity and diet are arguably mediators on the pathway between television exposure and overweight or obesity. For example, physical activity has been previously found to be an effect modifier of the association between television and obesity [19]. A fundamental principle of adjusting for a confounder is that the covariate is not an effect modifier or mediator of the relationship between exposure and outcome. Therefore, in our example adjusting for a mediator (e.g., physical activity) like a confounder may not result in the best estimate of the association [57]. Lastly, although an attempt was made to establish temporality, true cause and effect cannot be ascertained from this study design.

## Conclusion

The results from this analysis suggest that television exposure is associated with deleterious effects on physical activity, diet, and body weight status in adult participants of the ARIC cohort. Television exposure was associated cross-sectionally and prospectively with physical activity, diet and body weight. Consistent associations were observed in all analyses between higher television exposure and more unhealthy physical activity and dietary behaviors, but not body weight status. Adjusting for the baseline relationships attenuated, but did not eliminate, the prospective associations between physical activity and diet. These results support the hypothesis that television may be a substitute for time spent in more physically active pursuits and may contribute to both immediate and future dietary behaviors. Television may also play a significant role in body weight status and the burgeoning obesity epidemic. It is important for adults to recognize the amount of time spent in front of the television being sedentary may contribute to unhealthy lifestyles. Future research can help identify the behavioural correlates (e.g., physical activity, diet) of sedentary activity, and understand the combined impact on chronic disease.

## Abbreviations

TV: television; ARIC: Atherosclerosis Risk in Communities Study; BMI: body mass index; CI: confidence interval; FFQ: food frequency questionnaire; ANOVA: Analysis of Variance

## Competing interests

The authors declare that they have no competing interests.

## Authors' contributions

AMM and KRE developed the hypotheses. AMM carried out the literature review, statistical analysis, and drafted the manuscript. DC provided statistical consultation. GH and JS participated in the study design and collection of the ARIC data. AMM, KRE, MP, and JS provided contextual and epidemiologic expertise. All authors were involved in critical revisions of the manuscript and have read and approved the final manuscript.

## References

[B1] Robinson J, Godbey G (2005). Time in our hands. Futurist.

[B2] Robinson JP, Godbey G (2005). Busyness as usual. Social Research.

[B3] (2005). Television At A Glance.

[B4] Hu FB, Li TY, Colditz GA, Willett WC, Manson JE (2003). Television watching and other sedentary behaviors in relation to risk of obesity and type 2 diabetes mellitus in women. JAMA.

[B5] (2006). Nielsen Media Research Reports Television's Popularity is Still Growing.

[B6] FitzGerald SJ, Kriska AM, Pereira MA, DeCourten MP (1997). Associations among physical activity, television watching, and obesity in adult Pima Indians. Med Sci Sports Exerc.

[B7] Hu FB, Leitzmann MF, Stampfer MJ, Colditz GA, Willett WC, Rimm EB (2001). Physical activity and television watching in relation to risk for type 2 diabetes mellitus in men. Arch Intern Med.

[B8] Dunstan DW, Salmon J, Owen N, Armstrong T, Zimmet PZ, Welborn TA, Cameron AJ, Dwyer T, Jolley D, Shaw JE (2004). Physical activity and television viewing in relation to risk of undiagnosed abnormal glucose metabolism in adults. Diabetes Care.

[B9] Parsons TJ, Power C, Manor O (2006). Longitudinal physical activity and diet patterns in the 1958 British Birth Cohort. Med Sci Sports Exerc.

[B10] Bennett GG, Wolin KY, Viswanath K, Askew S, Puleo E, Emmons KM (2006). Television viewing and pedometer-determined physical activity among multiethnic residents of low-income housing. Am J Public Health.

[B11] Jakes R, Day N, Khaw K, Luben R, Oakes S, Welch A, Bingham S, Wareham N (2003). Television viewing and low participation in vigorous recreation are independently associated with obesity and markers of cardiovascular disease risk: EPIC-Norfolk population-based study. Eur J Clin Nutr.

[B12] Ching PL, Willett WC, Rimm EB, Colditz GA, Gortmaker SL, Stampfer MJ (1996). Activity level and risk of overweight in male health professionals. Am J Public Health.

[B13] Fung T, Hu F, Yu J, Chu N, Spiegelman D, Tofler G, Willett W, Rimm E (2000). Leisure-time physical activity, television watching, and plasma biomarkers ofobesity and cardiovascular disease risk. Am J Epidemiol.

[B14] Coakley E, Rimm E, Colditz G, Kawachi I, Willett W (1998). Predictors of weight change in men: Results from the Health Professionals Follow-Up Study. Intl J Obs Relat   Metab Disord.

[B15] Tucker L, Bagwell M (1991). Television viewing and obesity in adult females. Am J Public Health.

[B16] Tucker L, Friedman G (1989). Television viewing and obesity in adult males. Am J Public Health.

[B17] Jeffery RW, French SA (1998). Epidemic obesity in the United States: are fast foods and television viewing contributing?. Am J Public Health.

[B18] Buchowski MS, Sun M (1996). Energy expenditure, television viewing and obesity. Int J Obes Relat Metab Disord.

[B19] Salmon J, Bauman A, Crawford D, Timperio A, Owen N (2000). The association between television viewing and overweight among Australian adults participating in varying levels of leisure-time physical activity. Int J Obes Relat Metab Disord.

[B20] Kronenberg F, Pereira MA, Schmitz MK, Arnett DK, Evenson KR, Crapo RO, Jensen RL, Burke GL, Sholinsky P, Ellison RC, Hunt SC (2000). Influence of leisure time physical activity and television watching on atherosclerosis risk factors in the NHLBI Family Heart Study. Atherosclerosis.

[B21] Dunstan DW, Salmon J, Owen N, Armstrong T, Zimmet PZ, Welborn TA, Cameron AJ, Dwyer T, Jolley D, Shaw JE (2005). Associations of TV viewing and physical activity with the metabolic syndrome in Australian adults. Diabetologia.

[B22] Crawford DA, Jeffery RW, French SA (1999). Television viewing, physical inactivity and obesity. Int J Obes Relat Metab Disord.

[B23] Vioque J, Torres A, Quiles J (2000). Time spent watching television, sleep duration and obesity in adults living in Valencia, Spain. Int J Obes Relat Metab Disord.

[B24] Jackson R, Chambless L, Yang K, Byrne T, Watson R, Folsom A, Shahar E, Kalsbeek W (1996). Differences between respondents and nonrespondents in a multicenter community-based study vary by gender and ethnicity. J Clin Epidemiol.

[B25] Atherosclerosis Risk in Communities Investigators (1989). The Atherosclerosis Risk in Communities (ARIC) study: Design and objectives. Am J Epidemiol.

[B26] Baecke JA, Burema J, Frijters JE (1982). A short questionnaire for the measurement of habitual physical activity in epidemiological studies. Am J Clin Nutr.

[B27] Pereira MA, Folsom AR, McGovern PG, Carpenter M, Arnett DK, Liao D, Szklo M, Hutchinson RG (1999). Physical activity and incident hypertension in black and white adults: the Atherosclerosis Risk in Communities Study. Prev Med.

[B28] Evenson K, Rosamond W, Cai J, Toole J, Hutchinson R, Shahar E, Folsom A (1999). Physical activity and ischemic stroke risk: The Atherosclerosis Risk in Communities Study. Stroke.

[B29] Jacobs DR, Ainsworth BE, Hartman TJ, Leon AS (1993). A simultaneous evaluation of 10 commonly used physical activity questionnaires. Med Sci Sports Exerc.

[B30] Pols MA, Peeters PH, Bueno-De-Mesquita HB, Ocke MC, Wentink CA, Kemper HC, Collette HJ (1995). Validity and repeatability of a modified Baecke questionnaire on physical activity. Int J Epidemiol.

[B31] Philippaerts RM, Lefevre J (1998). Reliability and validity of three physical activity questionnaires in Flemish males. Am J Epidemiol.

[B32] Richardson MT, Ainsworth BE, Wu HC, Jacobs DR, Leon AS (1995). Ability of the Atherosclerosis Risk in Communities (ARIC)/Baecke Questionnaire to assess leisure-time physical activity. Int J Epidemiol.

[B33] Miller DJ, Freedson PS, Kline GM (1994). Comparison of activity levels using the Caltrac accelerometer and five questionnaires. Med Sci Sports Exerc.

[B34] Rauh MJ, Hovell MF, Hofstetter CR, Sallis JF, Gleghorn A (1992). Reliability and validity of self-reported physical activity in Latinos. Int J Epidemiol.

[B35] Gretbeck, Montoye (1990). Abstract #474. Med Sci Sports Exerc.

[B36] Mahoney, Freedson P (1990). Abstract #475. Med Sci Sports Exerc.

[B37] Cauley JA, LaPorte RE, Sandler RB, Schramm MM, Kriska AM (1987). Comparison of methods to measure physical activity in postmenopausal women. Am J Clin Nutr.

[B38] Philippaerts RM, Westerterp KR, Lefevre J (2001). Comparison of two questionnaires with a tri-axial accelerometer to assess physical activity patterns. Int J Sports Med.

[B39] Voorrips LE, Ravelli AC, Dongelmans PC, Deurenberg P, Van Staveren WA (1991). A physical activity questionnaire for the elderly. Med Sci Sports Exerc.

[B40] Philippaerts RM, Westerterp KR, Lefevre J (1999). Doubly labelled water validation of three physical activity questionnaires. Int J Sports Med.

[B41] Willett WC, Sampson L, Stampfer MJ, Rosner B, Bain C, Witschi J, Hennekens CH, Speizer FE (1985). Reproducibility and validity of a semiquantitative food frequency questionnaire. Am J Epidemiol.

[B42] Shimakawa T, Sorlie P, Carpenter MA, Dennis B, Tell GS, Watson R, Williams OD (1994). Dietary intake patterns and sociodemographic factors in the atherosclerosis risk in communities study. ARIC Study Investigators. Prev Med.

[B43] Houston DK, Stevens J, Cai J, Haines PS (2005). Dairy, fruit, and vegetable intakes and functional limitations and disability in a biracial cohort: the Atherosclerosis Risk in Communities Study. Am J Clin Nutr.

[B44] Subar AF, Thompson FE, Kipnis V, Midthune D, Hurwitz P, McNutt S, McIntosh A, Rosenfeld S (2001). Comparative Validation of the Block, Willett, and National Cancer Institute Food Frequency Questionnaires: The Eating at America's Table Study. Am J Epidemiol.

[B45] Eck LH, Klesges LM, Klesges RC (1996). Precision and estimated accuracy of two short-term food frequency questionnaires compared with recalls and records. J Clin Epidemiol.

[B46] Stevens J, Metcalf PA, Dennis BH, Tell GS, Shimakawa T, Folsom AR (1996). Reliability of a food frequency questionnaire by ethnicity, gender, age and education. Nutrition Research.

[B47] (1995). Physical status: The use and interpretation of anthropometry. Book Physical status: The use and interpretation of anthropometry.

[B48] Bouchard C (1999). Physical inactivity. Can J Cardiol.

[B49] Levine JA, Kotz CM (2005). NEAT – non-exercise activity thermogenesis – egocentric & geocentric environmental factors vs. biological regulation. Acta Physiol Scand.

[B50] Levine JA, Weg MWV, Hill JO, Klesges RC (2006). Non-exercise activity thermogenesis – The crouching tiger hidden dragon of societal weight gain. Arteriosclerosis Thrombosis and Vascular Biology.

[B51] Levine JA (2004). Non-exercise activity thermogenesis (NEAT). Nutr Rev.

[B52] Ward DS, Evenson KR, Vaughn A, Rodgers AB, Troiano RP (2005). Accelerometer use in physical activity: best practices and research recommendations. Med Sci Sports Exerc.

[B53] Bryant MJ, Lucove JC, Evenson KR, Marshall S (2006). Measurement of television viewing in children and adolescents: a systematic review. Obes Rev.

[B54] Hulens M, Vansant G, Claessens AL, Lysens R, Muls E (2003). Predictors of 6-minute walk test results in lean, obese and morbidly obese women. Scand J Med Sci Sports.

[B55] Feskanich D, Rimm EB, Giovannucci EL, Colditz GA, Stampfer MJ, Litin LB, Willett WC (1993). Reproducibility and validity of food intake measurements from a semiquantitative food frequency questionnaire. J Am Diet Assoc.

[B56] Rimm EB, Giovannucci EL, Stampfer MJ, Colditz GA, Litin LB, Willett WC (1992). Reproducibility and validity of an expanded self-administered semiquantitative food frequency questionnaire among male health professionals. Am J Epidemiol.

[B57] Bauman AE, Sallis JF, Dzewaltowski DA, Owen N (2002). Toward a better understanding of the influences on physical activity: the role of determinants, correlates, causal variables, mediators, moderators, and confounders. Am J Prev Med.

